# Supplementary material for: Electrochemical Immunosensor for the Quantification of S100B at Clinically Relevant Levels Using a Cysteamine Modified Surface
Source: Sensors (Basel). 2021 Mar 10;21(6):1929. doi: 10.3390/s21061929 (PMC8001999; doi:10.3390/s21061929)
Supplement: Supplementary file 1 [file sensors-21-01929-s001.pdf]

Supplementary Materials

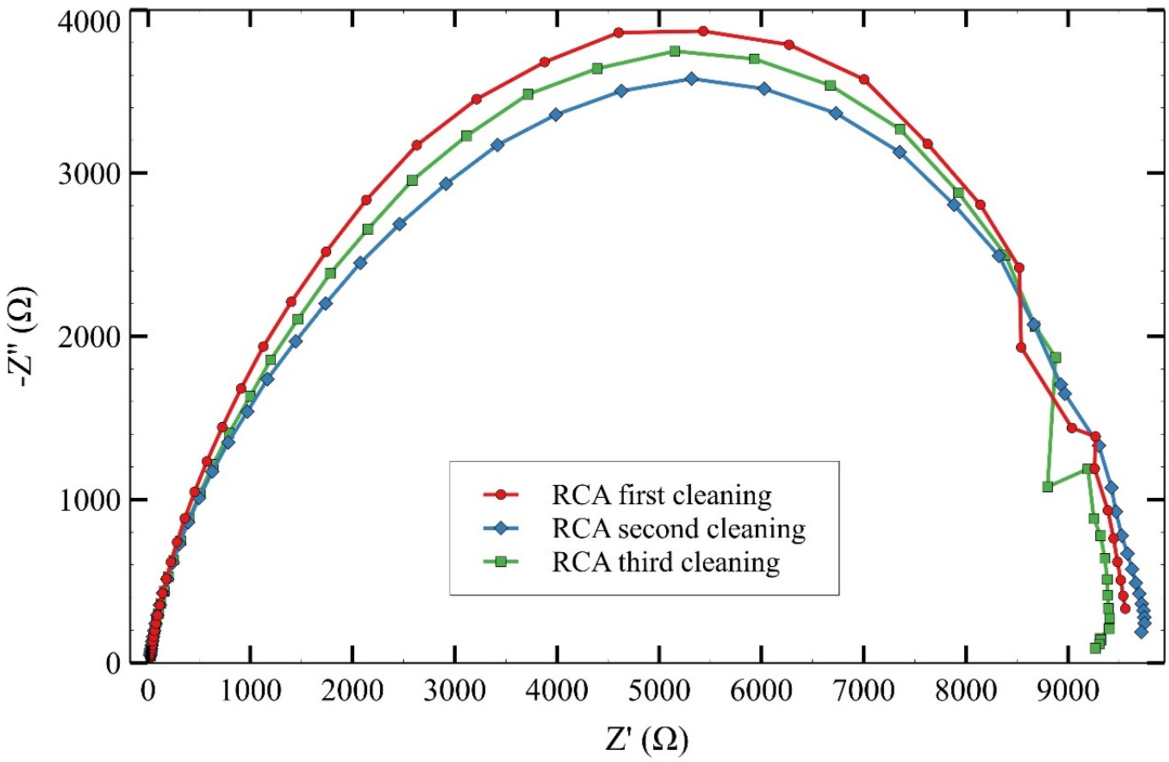

Figure S1. Nyquist plots for bare AuIDE after three successive RCA-1 cleanings.

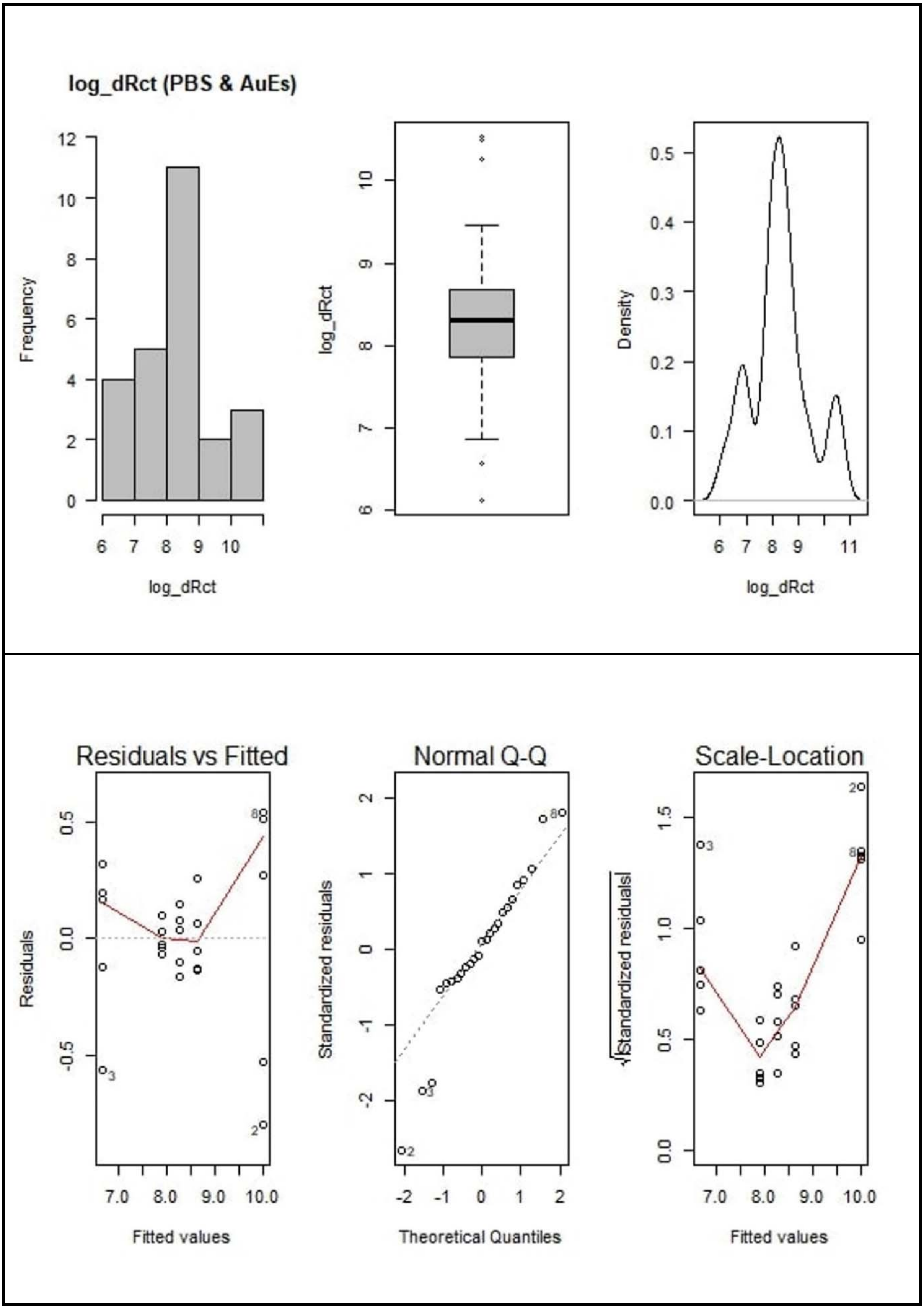

Figure S2. Graphically verification of assumptions of normality for AuEs-PBS dataset

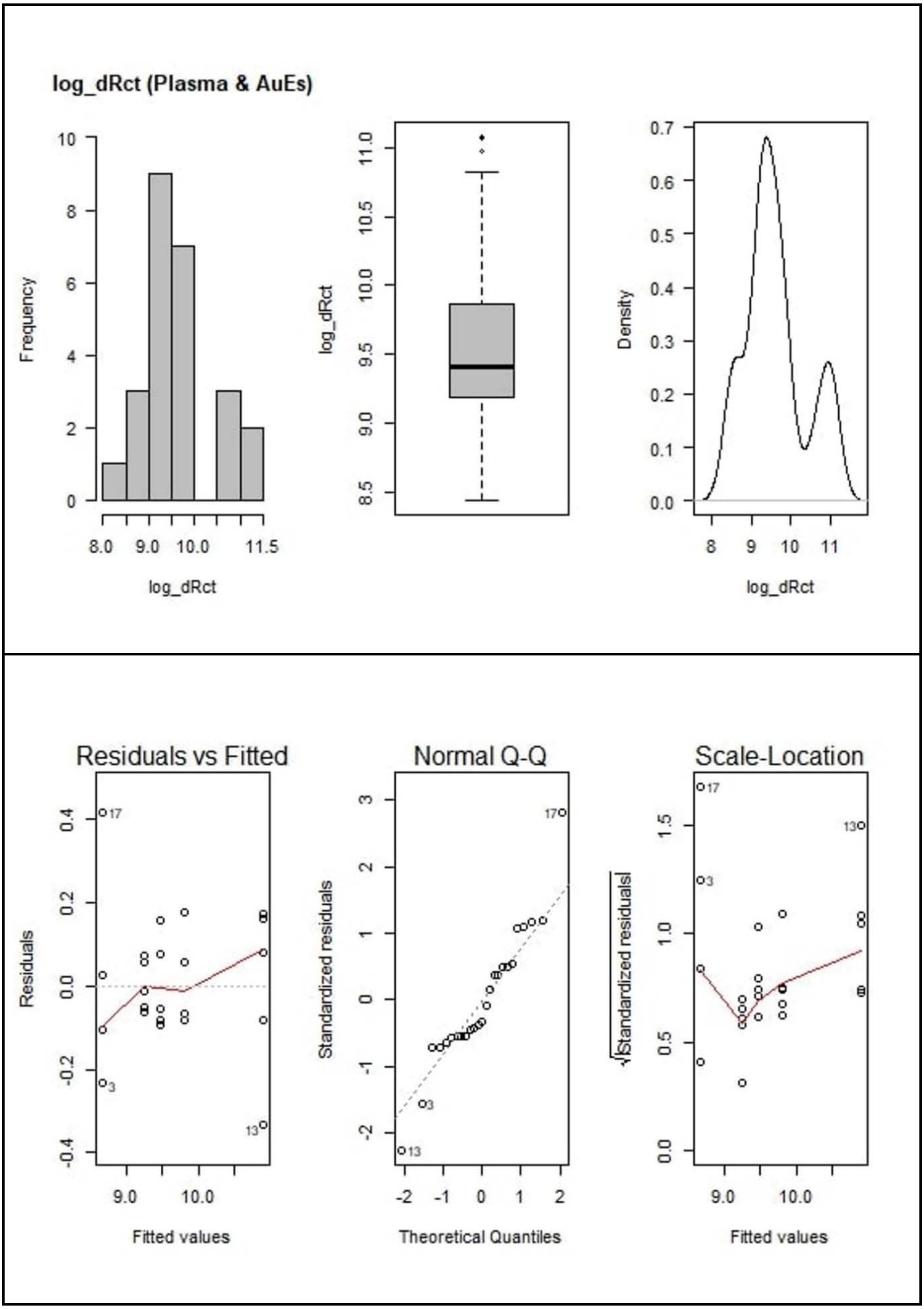

Figure S3. Graphically verification of assumptions of normality for AuES-plasma dataset

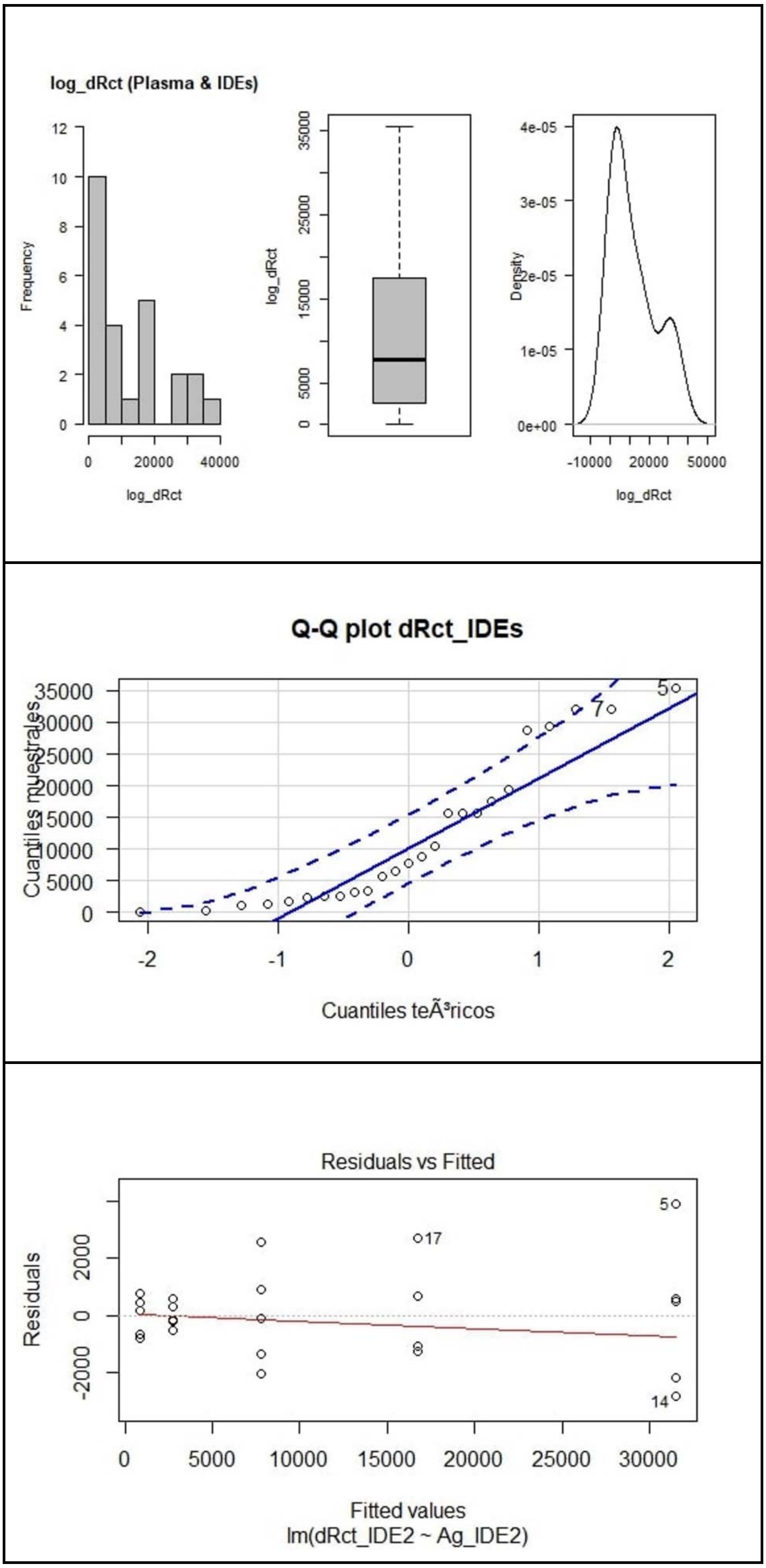

Figure S4. Graphically verification of assumptions of normality for AuIDES-plasma dataset

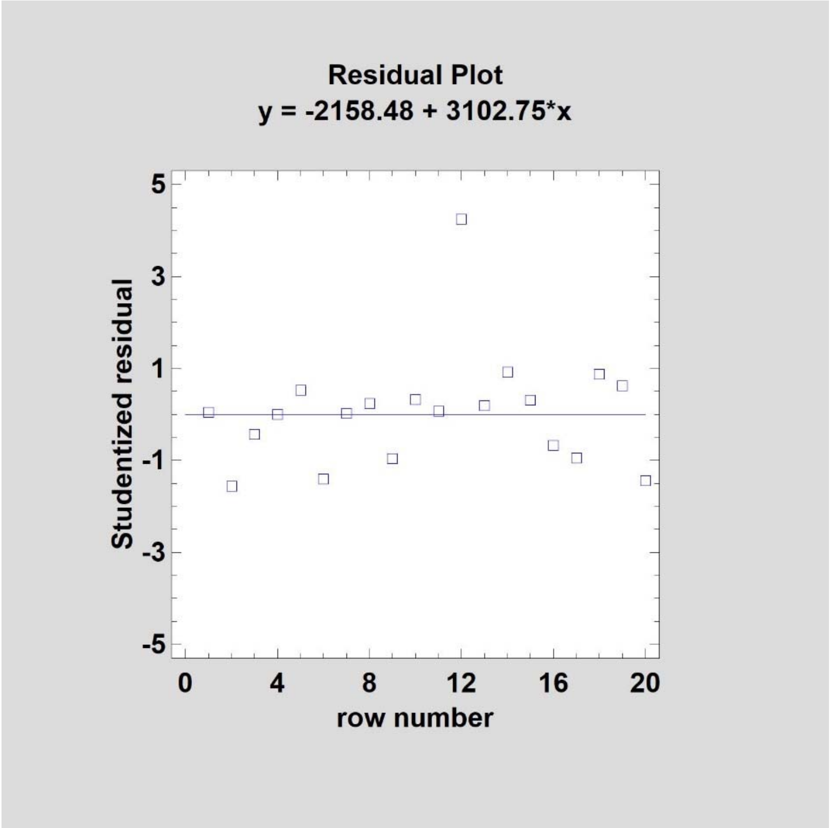

Figure S5. Analysis of the residuals structure for the AuEs-PBS regression model

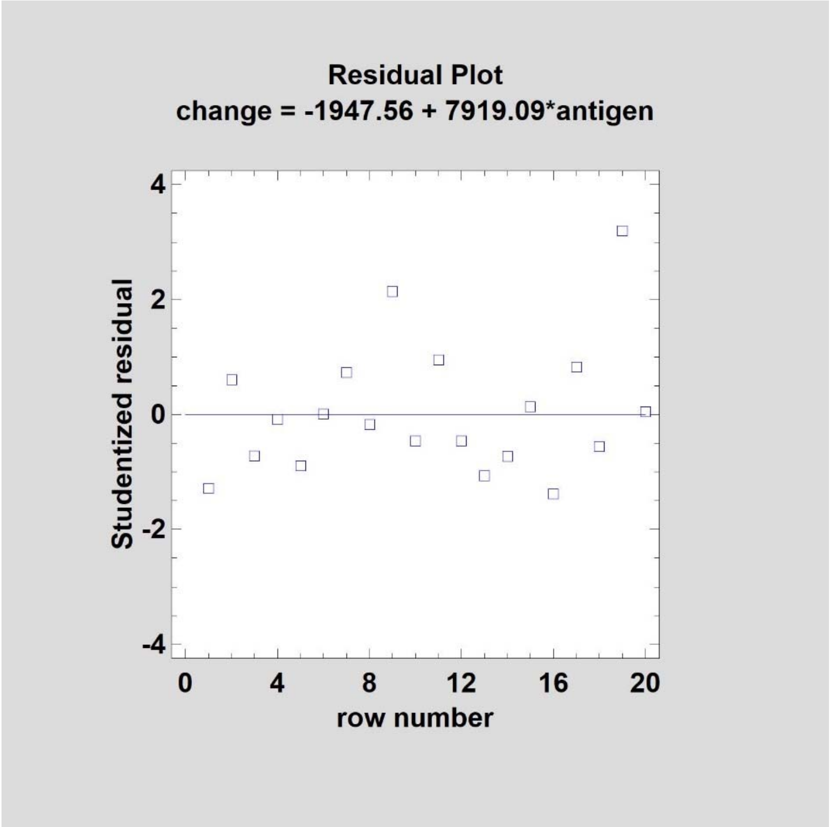

Figure S6. Analysis of the residuals structure for the AuEs-plasma regression model

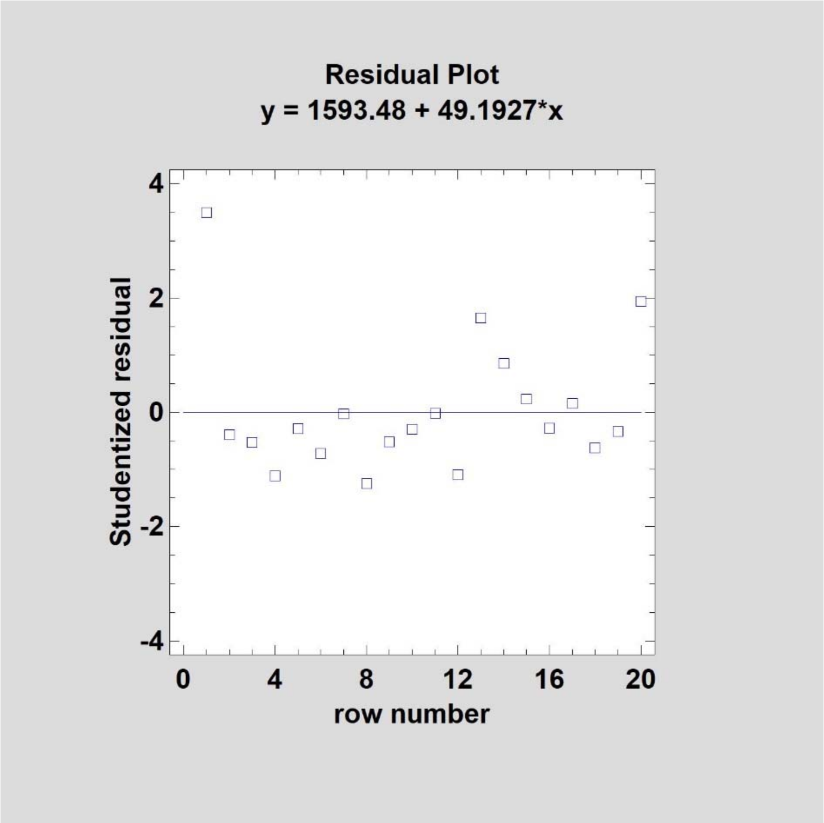

Figure S7. Analysis of the residuals structure for the AuIDEs-plasma regression model

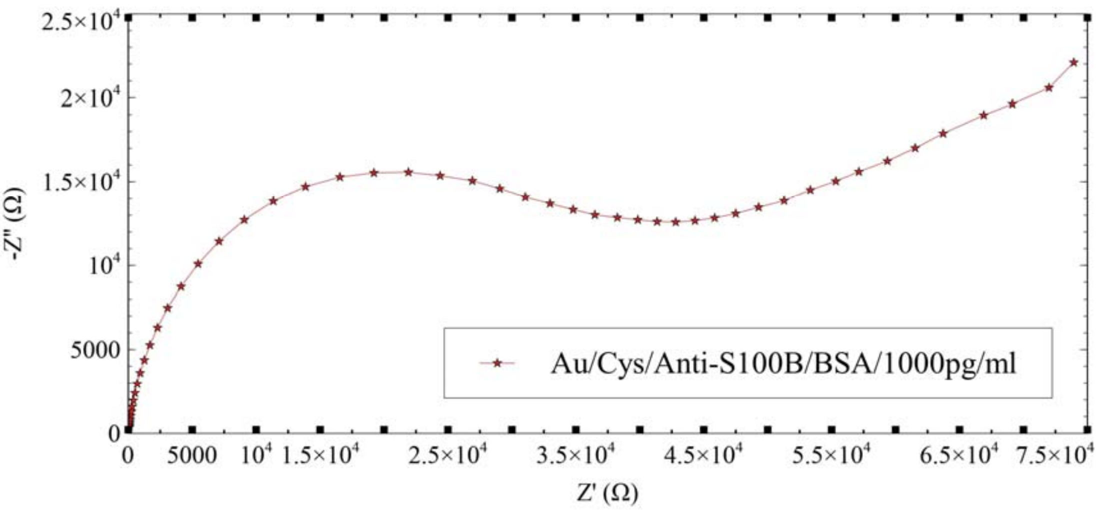

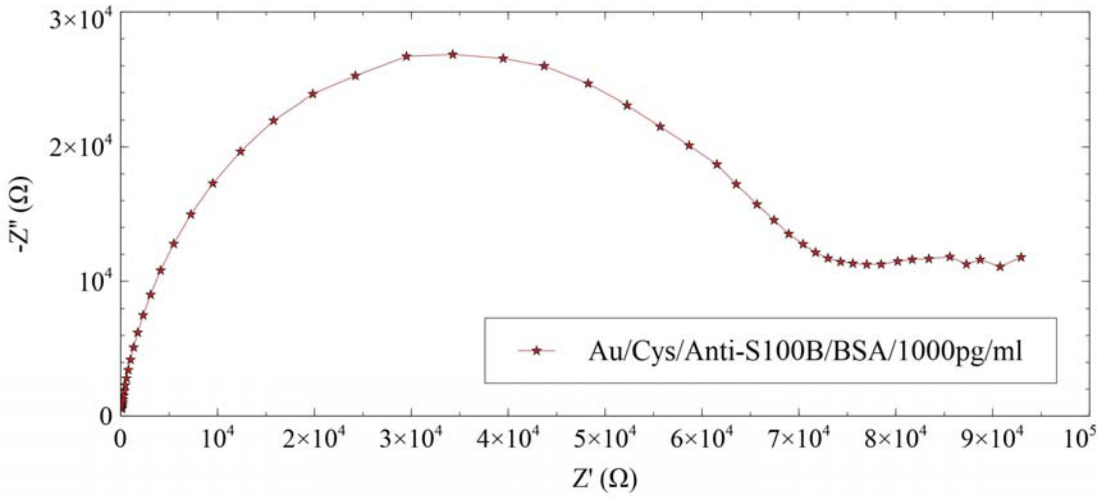

Figure S8. Nyquist plots for [S100B] 1,000pg/mL in AuEs-PBS (above) and AuEs-plasma (below).

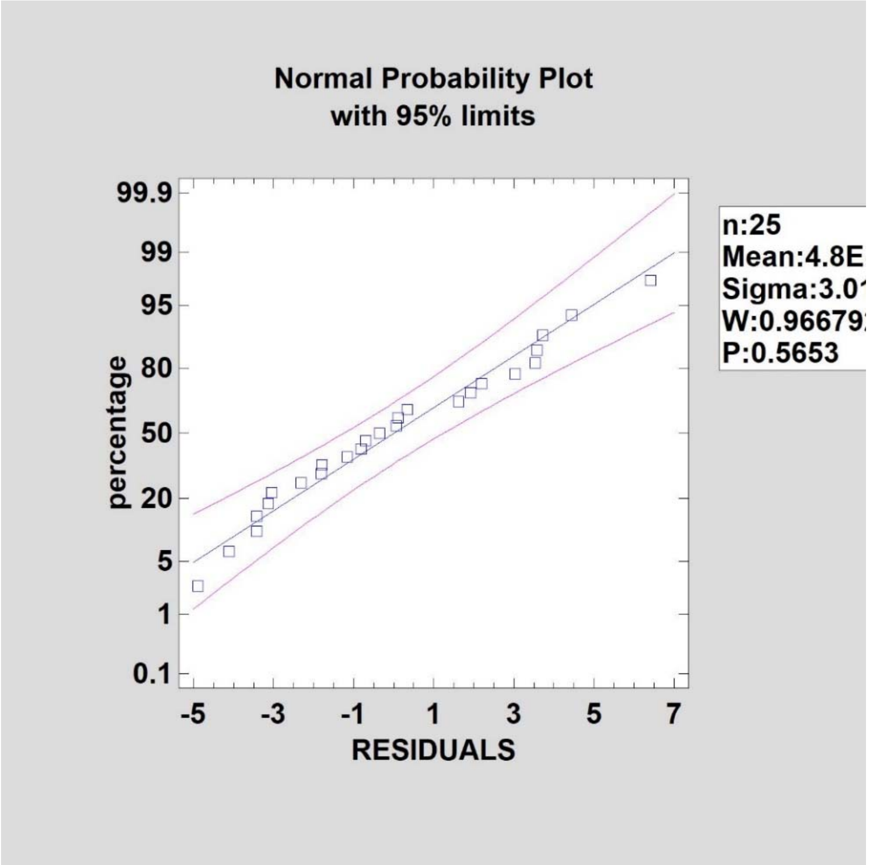

Figure S9. Normality of residuals for capacitance measurements in AuIDEs-plasma

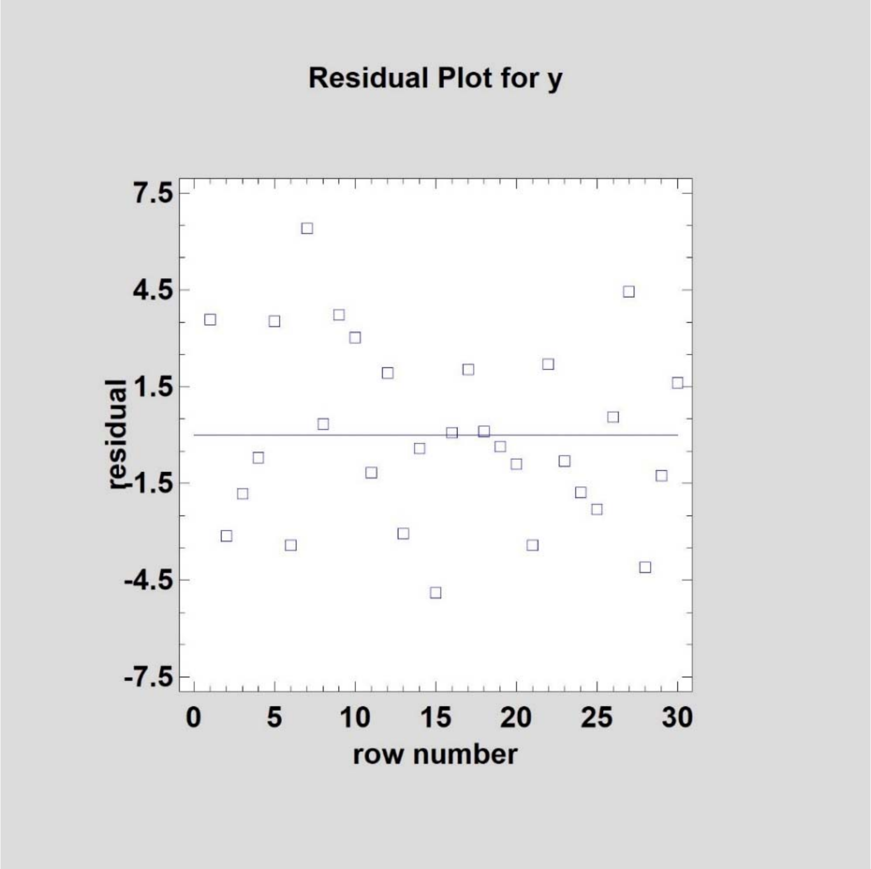

Figure S10. Independence of residuals for capacitance measurements in AuIDEs-plasma

Table S1. Analysis of variance and post-hoc test for AuEs-PBS data

```
# Normalization
> PBS$y = log(dRct_PBS)
> PBS$x = Ag_PBS

library(rstatix)
> ANOVA_PBS <- PBS %>% welch_anova_test(y ~ x)
> ANOVA_PBS
# A tibble: 1 x 7
  .y.       n statistic   DFn   DFd          p method
* <chr> <int>     <dbl> <dbl> <dbl>      <dbl> <chr>
1 y        25      45.3     4  9.16 0.00000496 Welch ANOVA
> summary(ANOVA_PBS)
     .y.                  n        statistic          DFn         DFd
 Length:1           Min.   :25   Min.   :45.29   Min.   :4   Min.   :9.158
 Class :character   1st Qu.:25   1st Qu.:45.29   1st Qu.:4   1st Qu.:9.158
 Mode  :character   Median :25   Median :45.29   Median :4   Median :9.158
                    Mean   :25   Mean   :45.29   Mean   :4   Mean   :9.158
                    3rd Qu.:25   3rd Qu.:45.29   3rd Qu.:4   3rd Qu.:9.158
                    Max.   :25   Max.   :45.29   Max.   :4   Max.   :9.158
       p               method
 Min.   :4.96e-06   Length:1
 1st Qu.:4.96e-06   Class :character
 Median :4.96e-06   Mode  :character
 Mean   :4.96e-06
 3rd Qu.:4.96e-06
 Max.   :4.96e-06
> #Prueba Post hoc
> #Games Howell PBS
> games_howell_test(PBS, y ~ Niveles, conf.level = 0.95, detailed = FALSE)
# A tibble: 10 x 8
   .y.   group1 group2 estimate conf.low conf.high    p.adj p.adj.signif
 * <chr> <chr>  <chr>     <dbl>    <dbl>     <dbl>    <dbl> <chr>
 1 y     10     31        1.21    0.514      1.90  0.006    **
 2 y     10     100       1.58    0.900      2.25  0.001    ***
 3 y     10     316       1.93    1.26       2.60  0.000293 ***
 4 y     10     1000      3.29    2.11       4.47  0.000215 ***
 5 y     31     100       0.368   0.126      0.610 0.007    **
 6 y     31     316       0.720   0.409      1.03  0.001    ***
 7 y     31     1000      2.08    0.855      3.31  0.008    **
 8 y     100    316       0.352   0.0257     0.678 0.035    *
 9 y     100    1000      1.72    0.503      2.93  0.014    *
10 y     316    1000      1.36    0.163      2.57  0.031    *
```

Table S2. Analysis of variance and post-hoc test for AuEs-plasma

```
#Normalization
> Plasma$y = log(dRct_Plasma)
> Plasma$x = Ag_Plasma

library(rstatix)
> ANOVA_Plasma <- Plasma %>% welch_anova_test(y ~ x)
> ANOVA_Plasma
# A tibble: 1 x 7
  .y.       n statistic   DFn   DFd           p method
* <chr> <int>     <dbl> <dbl> <dbl>       <dbl> <chr>
1 y        25      79.7     4  9.43 0.000000299 Welch ANOVA
> summary(ANOVA_Plasma)
     .y.                  n        statistic          DFn         DFd
 Length:1           Min.   :25   Min.   :79.73   Min.   :4   Min.   :9.431
 Class :character   1st Qu.:25   1st Qu.:79.73   1st Qu.:4   1st Qu.:9.431
 Mode  :character   Median :25   Median :79.73   Median :4   Median :9.431
                    Mean   :25   Mean   :79.73   Mean   :4   Mean   :9.431
                    3rd Qu.:25   3rd Qu.:79.73   3rd Qu.:4   3rd Qu.:9.431
                    Max.   :25   Max.   :79.73   Max.   :4   Max.   :9.431
       p               method
 Min.   :2.99e-07   Length:1
 1st Qu.:2.99e-07   Class :character
 Median :2.99e-07   Mode  :character
 Mean   :2.99e-07
 3rd Qu.:2.99e-07
 Max.   :2.99e-07
>
> #Prueba Post hoc
> ##Games Howell Plasma
> games_howell_test(Plasma, y ~ Niveles, conf.level = 0.95, detailed = FALSE)
# A tibble: 10 x 8
   .y.   group1 group2 estimate conf.low conf.high     p.adj p.adj.signif
 * <chr> <chr>  <chr>     <dbl>    <dbl>     <dbl>     <dbl> <chr>
 1 y     10     31        0.565   0.0822     1.05  0.028     *
 2 y     10     100       0.796   0.324      1.27  0.005     **
 3 y     10     316       1.14    0.664      1.61  0.000766  ***
 4 y     10     1000      2.22    1.71       2.73  0.0000033 ****
 5 y     31     100       0.231   0.0202     0.441 0.034     *
 6 y     31     316       0.570   0.353      0.787 0.000349  ***
 7 y     31     1000      1.66    1.25       2.07  0.000122  ***
 8 y     100    316       0.339   0.0927     0.586 0.009     **
 9 y     100    1000      1.43    1.03       1.83  0.0000671 ****
10 y     316    1000      1.09    0.686      1.49  0.000298  ***
```

Table S3. Analysis of variance and post-hoc test for AuIDEs-plasma

```
> #Welch ANOVA IDEs
> library(rstatix)
> ANOVAw <- PBS %>% welch_anova_test(dRct_IDE2 ~ Ag_IDE2)
> ANOVAw
# A tibble: 1 x 7
  .y.           n statistic   DFn   DFd             p method
* <chr>     <int>     <dbl> <dbl> <dbl>         <dbl> <chr>
1 dRct_IDE2    25      199.     4  9.18 0.00000000661 Welch ANOVA
> summary(ANOVAw)
     .y.                  n        statistic         DFn         DFd
 Length:1           Min.   :25   Min.   :199.4   Min.   :4   Min.   :9.18
 Class :character   1st Qu.:25   1st Qu.:199.4   1st Qu.:4   1st Qu.:9.18
 Mode  :character   Median :25   Median :199.4   Median :4   Median :9.18
                    Mean   :25   Mean   :199.4   Mean   :4   Mean   :9.18
                    3rd Qu.:25   3rd Qu.:199.4   3rd Qu.:4   3rd Qu.:9.18
                    Max.   :25   Max.   :199.4   Max.   :4   Max.   :9.18
       p               method
 Min.   :6.61e-09   Length:1
 1st Qu.:6.61e-09   Class :character
 Median :6.61e-09   Mode  :character
 Mean   :6.61e-09
 3rd Qu.:6.61e-09
 Max.   :6.61e-09

library(rstatix)
#ANOVA_Plasma <- Plasma %>% welch_anova_test(y ~ x)
ANOVA_Plasma <- qf1v02 %>% welch_anova_test(Rct_change ~ Log_S100B)
ANOVA_Plasma
summary(ANOVA_Plasma)
games_howell_test(qf1v02, dRct ~ Niveles, conf.level = 0.95, detailed = FALSE)

# A tibble: 10 x 8
   .y.   group1 group2 estimate conf.low conf.high      p.adj p.adj.signif
 * <chr> <chr>  <chr>     <dbl>    <dbl>     <dbl>      <dbl> <chr>
 1 dRct  1      1.5       1278.     445.     2111. 0.006      **
 2 dRct  1      2         6312     2731.     9893. 0.006      **
 3 dRct  1      2.5      15287.   11959.    18615. 0.000134   ***
 4 dRct  1      3        30003.   24731.    35275. 0.0000883  ****
 5 dRct  1.5    2         5034.    1503.     8565. 0.013      *
 6 dRct  1.5    2.5      14009.   10732.    17287. 0.000112   ***
 7 dRct  1.5    3        28726.   23494.    33957. 0.0000799  ****
 8 dRct  2      2.5       8975.    5126.    12824. 0.000292   ***
 9 dRct  2      3        23691.   18538.    28844. 0.00000461 ****
10 dRct  2.5    3        14716.    9614.    19818. 0.000132   ***
```

Table S4. ANOVA of AuEs-PBS regression model

Coefficients

Parameter	Least Squares Estimate	Standard Error	T Statistic	P-Value
Intercept	-2158.48	405.799	-5.31909	0.0000
Slope	3102.75	220.889	14.0467	0.0000

Analysis of Variance

Source	Sum of Squares	Df	Mean Square	F-Ratio	P-Value
Model	6.0169E7	1	6.0169E7	197.31	0.0000
Residual	5.48908E6	18	304949.		
Total (Corr.)	6.56581E7	19			

Correlation Coefficient = 0.957287
R-squared = 91.6399 percent
R-squared (adjusted for d.f.) = 91.1755 percent
Standard Error of Est. = 552.222
Mean absolute error = 379.384
Durbin-Watson statistic = 1.92298 (P=0.4467)
Lag 1 residual autocorrelation = -0.00801953

Analysis of Variance with Lack-of-Fit

Source	Sum of Squares	Df	Mean Square	F-Ratio	P-Value
Model	6.0169E7	1	6.0169E7	197.31	0.0000
Residual	5.48908E6	18	304949.		
Lack-of-Fit	340883.	2	170441.	0.53	0.5987
Pure Error	5.1482E6	16	321762.		
Total (Corr.)	6.56581E7	19			

Table S5. ANOVA of AuEs-plasma regression model

Coefficients

Parameter	Least Squares Estimate	Standard Error	T Statistic	P-Value
Intercept	-1947.55	1174.72	-1.65789	0.1147
Slope	7919.07	639.435	12.3845	0.0000

Analysis of Variance

Source	Sum of Squares	Df	Mean Square	F-Ratio	P-Value
Model	3.91948E8	1	3.91948E8	153.38	0.0000
Residual	4.59987E7	18	2.55549E6		
Total (Corr.)	4.37946E8	19			

Correlation Coefficient = 0.946027
R-squared = 89.4967 percent
R-squared (adjusted for d.f.) = 88.9132 percent
Standard Error of Est. = 1598.59
Mean absolute error = 1186.34
Durbin-Watson statistic = 2.44609 (P=0.8419)
Lag 1 residual autocorrelation = -0.264542

Analysis of Variance with Lack-of-Fit

Source	Sum of Squares	Df	Mean Square	F-Ratio	P-Value
Model	3.91948E8	1	3.91948E8	153.38	0.0000
Residual	4.59987E7	18	2.55549E6		
Lack-of-Fit	5.44701E6	2	2.72351E6	1.07	0.3648
Pure Error	4.05517E7	16	2.53448E6		
Total (Corr.)	4.37946E8	19			

Table S6. ANOVA of AuIDEs-plasma regression model

Coefficients

Parameter	Least Squares Estimate	Standard Error	T Statistic	P-Value
Intercept	1593.48	441.714	3.6075	0.0020
Slope	49.1927	2.6526	18.5451	0.0000

Analysis of Variance

Source	Sum of Squares	Df	Mean Square	F-Ratio	P-Value
Model	7.10305E8	1	7.10305E8	343.92	0.0000
Residual	3.71758E7	18	2.06532E6		
Total (Corr.)	7.47481E8	19			

Correlation Coefficient = 0.974815
R-squared = 95.0265 percent
R-squared (adjusted for d.f.) = 94.7502 percent
Standard Error of Est. = 1437.12
Mean absolute error = 1011.55
Durbin-Watson statistic = 1.41317 (P=0.0953)
Lag 1 residual autocorrelation = 0.0210756

Analysis of Variance with Lack-of-Fit

Source	Sum of Squares	Df	Mean Square	F-Ratio	P-Value
Model	7.10305E8	1	7.10305E8	343.92	0.0000
Residual	3.71758E7	18	2.06532E6		
Lack-of-Fit	1.13514E7	2	5.6757E6	3.52	0.0542
Pure Error	2.58244E7	16	1.61402E6		
Total (Corr.)	7.47481E8	19			

Table S7. Experimental design results for capacitance measurements in AuIDEs-plasma

Run	Log S100B	Capacitance increment (%)
1	1.5	20.6328
2	2.5	49.1726
3	3	61.6102
4	2.5	51.606
5	2.5	55.8388
6	2.5	48.8803
7	2	36.6536
8	1	9.19385
9	2.5	56.0243
10	1	11.8846
11	1	7.69152
12	2	32.1593
13	1.5	14.0025
14	0	1.20397
15	3	58.5319
16	3	63.4864
17	0	3.64881

18	1	8.95861
19	1.5	16.7007
20	0	0.712611
21	2	26.8224
22	3	65.6225
23	2	29.4288
24	1.5	15.2647
25	1	6.54384
26	0	2.17192
27	3	67.86
28	2	26.1342
29	0	0.353168
30	1.5	18.6712

Table S8. Verification of homoscedasticity for capacitance measurements in AuIDEs-plasma

Variance Check

	Test	P-Value
Levene's	1.75958	0.1595

Comparison	Sigma1	Sigma2	F-Ratio	P-Value
0 / 1	1.32495	1.99995	0.438896	0.4447
0 / 1.5	1.32495	2.64864	0.250237	0.2083
0 / 2	1.32495	4.30156	0.094874	0.0424
0 / 2.5	1.32495	3.47671	0.145232	0.0883
0 / 3	1.32495	3.59645	0.135722	0.0789
1 / 1.5	1.99995	2.64864	0.570153	0.5996
1 / 2	1.99995	4.30156	0.216165	0.1671
1 / 2.5	1.99995	3.47671	0.330903	0.3094
1 / 3	1.99995	3.59645	0.309235	0.2820
1.5 / 2	2.64864	4.30156	0.379136	0.3703
1.5 / 2.5	2.64864	3.47671	0.580376	0.6111
1.5 / 3	2.64864	3.59645	0.542373	0.5680
2 / 2.5	4.30156	3.47671	1.53079	0.6900
2 / 3	4.30156	3.59645	1.43055	0.7371
2.5 / 3	3.47671	3.59645	0.934519	0.9492

Table S9. ANOVA and post-hoc test for capacitance measurements in AuIDEs-plasma

Source	Sum of Squares	Df	Mean Square	F-Ratio	P-Value
Between groups	15138.9	5	3027.79	322.70	0.0000
Within groups	225.184	24	9.38266		
Total (Corr.)	15364.1	29			

Multiple Range Tests

Method: 95.0 percent Tukey HSD

x	Count	Mean	Homogeneous Groups					
0	5	1.6181	X					
1	5	8.85448		X				
1.5	5	17.0544			X			
2	5	30.2397				X		
2.5	5	52.3044					X	
3	5	63.4222						X

Contrast	Sig.	Difference	+/- Limits
0 - 1	*	-7.23639	5.99125
0 - 1.5	*	-15.4363	5.99125
0 - 2	*	-28.6216	5.99125
0 - 2.5	*	-50.6863	5.99125
0 - 3	*	-61.8041	5.99125
1 - 1.5	*	-8.1999	5.99125
1 - 2	*	-21.3852	5.99125
1 - 2.5	*	-43.4499	5.99125
1 – 3	*	-54.5677	5.99125
1.5 - 2	*	-13.1853	5.99125
1.5 - 2.5	*	-35.25	5.99125
1.5 - 3	*	-46.3678	5.99125
2 - 2.5	*	-22.0647	5.99125
2 – 3	*	-33.1825	5.99125
2.5 - 3	*	-11.1178	5.99125

* denotes a statistically significant difference.

Table S10. Assessment of the reproducibility of the response variable (ΔRct) in AuEs-PBS, AuEs-plasma and AuIDEs-plasma through the estimation of the relative standard deviation (RSD).

ΔRct **AuEs-PBS**			
Log[S100B]	**mean**	**SD**	**RSD**
1	845	231.6	27.4
1.5	2704.8	166.7	6.2
2	3926.4	443.0	12.6
2.5	5609	867.3	15.5
3	24942.6	11607.9	46.5

ΔRct **AuEs Plasma**			
Log[S100B]	**mean**	**SD**	**RSD**
1	6006.8	1492.1	24.8
1.5	10309.2	569.6	5.5
2	13028	1328.8	11.4
2.5	18298.6	1947.6	10.6
3	54989.8	9664.6	17.6

ΔRct **AuIDEs Plasma**			
Log[S100B]	**mean**	**SD**	**RSD**
1	1494.6	244.2	16.3
1.5	2772.2	390.0	14.1
2	7806.6	1629.0	23.3
2.5	16781.6	1516.4	9.0
3	31497.8	2384.7	7.6

Table S11. Assessment of the reproducibility of the single frequency analysis using in AuIDEs through the estimation of the relative standard deviation (RSD).

Log[S100B]	%Change in Cdl*	mean	SD	RSD
1	9.0	8.9	2.0	22.6
	9.2			
	7.7			
	11.9			
	6.5			
1.5	18.7	30.2	4.3	14.2
	14.0			
	20.6			
	15.3			
	16.7			
2	26.8	7806.6	1629.0	23.3
	32.2			
	29.4			
	36.7			
	26.1			
2.5	56.0	16781.6	1516.4	9.0
	51.6			
	49.2			
	48.9			
	55.8			
3	61.6	63.4	3.6	5.7
	58.5			
	65.6			
	67.9			
	63.5			

*Double-layer capacitance
